# From pole parameters to line shapes and branching ratios

**DOI:** 10.1140/epjc/s10052-024-12884-6

**Published:** 2024-06-10

**Authors:** L. A. Heuser, G. Chanturia, F.-K. Guo, C. Hanhart, M. Hoferichter, B. Kubis

**Affiliations:** 1 Forschungszentrum Jülich, Institute for Advanced Simulation, 52425 Jülich, Germany; 2 Helmholtz-Institut für Strahlen- und Kernphysik and Bethe Center for Theoretical Physics, Universität Bonn, 53115 Bonn, Germany; 3 CAS Key Laboratory of Theoretical Physics, Institute of Theoretical Physics, Chinese Academy of Sciences, Beijing, 100190 China; 4 School of Physical Sciences, University of Chinese Academy of Sciences, Beijing, 100049 China; 5 Peng Huanwu Collaborative Center for Research and Education, Beihang University, Beijing, 100191 China; 6 Albert Einstein Center for Fundamental Physics, Institute for Theoretical Physics, University of Bern, Sidlerstrasse 5, 3012 Bern, Switzerland

## Abstract

Resonances are uniquely characterized by their complex pole locations and the corresponding residues. In practice, however, resonances are typically identified experimentally as structures in invariant mass distributions, with branching fractions of resonances determined as ratios of count rates. To make contact between these quantities it is necessary to connect line shapes and resonance parameters. In this work we propose such a connection and illustrate the formalism with detailed studies of the $\rho (770)$ and $f_0(500)$ resonances. Based on the line shapes inferred from the resonance parameters along these lines, expressions for partial widths and branching ratios are derived and compared to other approaches in the literature.

## Introduction

Most hadronic states are not stable in quantum chromodynamics (QCD) and possess a decay width too large to be approximated by a pole on the real axis. Instead, such resonances are described mathematically by poles in the complex-energy plane, and their characterization therefore requires an analytic continuation of the scattering matrix. In this way, the coupling of a resonance to a given decay channel is determined by the residue, that is, the strength of the scattering amplitude at the resonance pole. In practice, however, this analytic continuation can be highly non-trivial, and the projection onto the real axis, where experiments are performed, can differ widely depending on the complexity of the system. This ranges from clear-cut cases such as the $\rho (770)$, in which the resonance peak is clearly visible in the cross section, via examples such as the $f_0(500)$, in which case only a broad bump is observed, to complicated multi-channel systems such as the $f_0(980)$, which may show up as a narrow peak or a dip structure. In the last example, the line shape can differ so dramatically depending on the source that drives its production, since this is what controls the interference pattern of the given resonance with its background. It is therefore not at all straightforward to experimentally define branching ratios for a resonance. Even in cases in which the resonance pole parameters can be determined reliably via dispersive analyses of the scattering matrix, the concept of branching ratios needs to be understood theoretically in terms of pole parameters. The main goal of this work is to establish such a connection.

The framework we propose here is based on the two-potential formalism [1], constructed in such a way that constraints from analyticity and unitarity are maintained, while allowing for enough freedom to parameterize the effects of left-hand cuts (LHCs). To argue that such an approach constitutes, in fact, a minimal solution to the general problem, we proceed as follows. After defining the formalism in Sect. 2 for *S*-waves and discussing its generalization to higher partial waves in Sect. 3, with some details on conventions relegated to Appendices A and B, we start in Sect. 4 with the application to the $\rho (770)$, a resonance structure so clear that even the Breit–Wigner ansatz [2] gives a reasonable description. As a first step to improve beyond such a model, self-energy corrections need to be included to restore the correct analyticity properties, which leads to a form closely resembling the Gounaris–Sakurai parameterization of the $\rho (770)$ [3]. However, we will show that with this procedure only real and imaginary parts of the pole location,

1.1 $$\begin{aligned} \sqrt{s_\text {R}} = M_\text {R}-i\frac{\varGamma _\text {R}}{2}\, , \end{aligned}$$

by convention expressed in terms of the pole mass $M_\text {R}$ and pole width $\varGamma _\text {R}$, can be reproduced exactly, while the residue is predicted in terms of these parameters. For a high-precision description of pion–pion ($\pi \pi$) scattering [4–8] and the resulting $\rho (770)$ pole parameters obtained from analytic continuation of the Roy equations [9], this does not provide sufficient flexibility. Reproducing the $\rho (770)$ parameters at the precision level is not only important to illustrate how our formalism works, but also of phenomenological interest, as a starting point to describe $4\pi$ inelasticities in the electromagnetic form factor of the pion [10, 11], which is critical for a better understanding of tensions in the $2\pi$ contribution to hadronic vacuum polarization [8, 12, 13].

Next in complexity we turn to the $f_0(500)$ in Sect. 5. While the existence of this lowest-lying resonance in QCD was contested for decades [14], the required analytic continuation deep into the complex plane can again be performed in a reliable manner based on dispersion relations [15–17], despite the fact that the $f_0(500)$ is not visible in the $\pi \pi$ *S*-wave phase shift as a clear resonance structure (the same is true for scalar form factors, see, e.g., Refs. [18–21]). Moreover, in this case the presence of an Adler zero [22, 23] is critical to obtain a realistic line shape. For instance, it is known from the inverse-amplitude method [24, 25] that unitarizing amplitudes from chiral perturbation theory (ChPT) with the right Adler zero, the $f_0(500)$ parameters are reproduced with reasonable accuracy. Here, we will show the opposite direction, finding that starting from the $f_0(500)$ resonance parameters, our formalism automatically produces an Adler zero in the vicinity of its ChPT expectation. We also detail how the correct threshold behavior of the LHCs can be incorporated, see Appendix C, and evaluate higher-order chiral corrections to the Adler zero, see Appendix D.

Having demonstrated how our formalism recovers the $\rho (770)$ and $f_0(500)$ as resonances in $\pi \pi$ scattering, we turn to the generalization to multi-channel systems in Sect. 6. In such a case, if a resonance couples to various channels, the imaginary part of the pole position acquires contributions from all of them. Depending on the Riemann sheet on which the most significant pole is located, the individual imaginary parts not necessarily add, and some care is required in defining consistent branching fractions and decay widths, see, e.g., Refs. [26–28] for recent works in this direction. While usually the problem is phrased as the determination of pole parameters from the analytic continuation of scattering amplitudes [29], we take here the opposite perspective and discuss to what extent line shapes, and from those branching ratios of resonances, can be deduced from a set of pole parameters. The main goal is to replace common prescriptions to turn residues into branching fractions by a better justified recipe. For example, a narrow-width formula for the $f_0(500)\rightarrow \gamma \gamma$ decay [17, 30–32] fails to account for the complicated line shape of the $f_0(500)$, while the branching ratio for $f_0(500)\rightarrow \bar{K} K$ [33] would even vanish, since the resonance mass lies below the $\bar{K} K$ threshold. Instead, we will show how our formalism allows us to derive well-defined, normalized spectral functions, from which partial widths and branching ratios can be inferred in a consistent manner. As test cases, we again consider $\rho (770)$ and $f_0(500)$, comparing our prescription to other proposals in the literature. Our formalism can be generalized to more complicated cases such as the $f_0(980)$ [16, 17, 34] or $a_0(980)$ [35, 36], for which different Riemann sheets play a role. In Sect. 7 we summarize our main results and give an outlook towards such future applications.

## *S*-wave formalism

### Scattering amplitude and residues

All information on a scattering process is encoded in the scattering amplitude $\mathcal M$, connected to the *S*-matrix via

2.1 $$\begin{aligned}&_{\text {out}}\phantom{a}{\langle {p_{1}' p_{2}',b}|} \mathcal {S} - 1 {|{p_1 p_2,a}\rangle }_\text {in}\nonumber&\\&\quad = i(2\pi )^4\delta ^4(p_1+p_2-p_{1}'-p_{2}')\,\mathcal {M}_{ba} \, , \end{aligned}$$

where, for concreteness, we concentrate on a two-to-two reaction. Close to the resonance pole it can be expanded into a Laurent series as

2.2 $$\begin{aligned} \mathcal {M}_{ba} = - \frac{\mathcal {R}_{ba}}{s-s_\text {R}} + \text{ regular } \text{ terms } \, , \end{aligned}$$

where *a* and *b* are channel indices. The residue $\mathcal {R}_{ba}$ can be conveniently extracted from the amplitude via

2.3 $$\begin{aligned} \mathcal {R}_{ba} = -\frac{1}{2\pi i}\oint ds \, \mathcal {M}_{ba} \, , \end{aligned}$$

where the closed integration path needs to be chosen such that it runs counterclockwise and the pole of interest is the only non-analyticity enclosed. The factorization of the residue $(\mathcal {R}_{ba})^2 = \mathcal {R}_{aa}\times \mathcal {R}_{bb}$ allows one to introduce pole couplings according to

2.4 $$\begin{aligned} \tilde{g}_a = H(s_p)\mathcal {R}_{ba} /\sqrt{\mathcal {R}_{bb}} \, . \end{aligned}$$

The function $H(s_p)$ is introduced here to collect convention-dependent factors often introduced for the effective couplings, e.g., for higher partial waves $H(s_p)$ traditionally absorbs the centrifugal barrier factor. The conventions relevant for the effective couplings employed in this work are provided in Appendix A. It should be stressed that these pole couplings are the only model- and reaction-independent quantities that allow one to quantify the transition strength of a given resonance to some channel *a*.

### Dyson series and self energy

As a starting point, we consider the case of a resonance coupling to a single continuum channel in an *S*-wave. Higher partial waves are discussed in Sect. 3 and the generalization to more channels is provided in Sect. 6, where also partial widths and branching ratios are introduced. Theoretically, the physical propagator of a single resonance, *G*(*s*), emerges as the solution of the Dyson equation for some given self-energy function $\varSigma (s)$^1^:

2.5 $$\begin{aligned} G(s) = G_0(s)-G_0(s)g^2\varSigma (s)G(s)\, , \end{aligned}$$

with the bare propagator

2.6 $$\begin{aligned} G_0(s)=(s-m^2)^{-1}\, . \end{aligned}$$

Equation (2.5) is solved by

2.7 $$\begin{aligned} G(s) = \big (s-m^2+g^2\varSigma (s)\big )^{-1} \, . \end{aligned}$$

Unitarity requires both *g* and *m* to be real parameters. The self energy $\varSigma (s)$ contains all one-particle irreducible diagrams with respect to the studied resonance that contribute to the two-point function in the resonance channel.

In the simplest scenario in which there is no background term and the complete interaction of the scattering particles is provided by the resonance one has

2.8 $$\begin{aligned} \text {disc}\,\varSigma (s) = 2i\rho (s) \, , \end{aligned}$$

where

2.9 $$\begin{aligned} \rho (s)=\frac{1}{16\pi }\frac{2q}{\sqrt{s}}\, , \qquad q=\frac{1}{2}\sqrt{s-4M^2} \, , \end{aligned}$$

*M* is the mass of the particles in the continuum channel, and *q* denotes the momentum of the outgoing particles in the center-of-mass frame. In this work we mostly study channels with particles of equal mass, however, the generalization to different masses is straightforward. In case of absence of a background term, such that the discontinuity is provided by Eq. (2.8), the self energy $\varSigma (s)$ equals the polarization function $\varPi (s)$, which can be written as a once-subtracted dispersion integral

2.10 $$\begin{aligned} \varPi (s) = b + \frac{s-s_0}{\pi }\int _{s_\text {thr}}^\infty \frac{\text {d}s'}{s'-s_0}\frac{\rho (s')}{s'-s} = b + \varPi ^r(s) \, , \end{aligned}$$

with some subtraction constant *b* that can be absorbed into other parameters of the amplitude. The scattering threshold $4M^2$ is denoted as $s_\text {thr}$, and $s_0$ is the subtraction point. The index *r* indicates that $\varPi ^r(s)$ is the renormalized self energy. Since from now on all self energies are renormalized, we drop the index *r* again to ease notation. For $s_0=4M^2$ one finds

2.11 $$\begin{aligned} \varPi (s)=\frac{\rho (s)}{\pi }\log \left( \frac{16\pi \rho (s)-1}{16\pi \rho (s)+1}\right) \end{aligned}$$

for all values of *s* on the first sheet. Under these conditions the scattering amplitude reads

2.12 $$\begin{aligned} \mathcal {M}(s)=-\frac{g^2}{s-m^2+g^2\varPi (s)} \, . \end{aligned}$$

To obtain the correct resonance pole location of $\mathcal {M}$, one therefore has to demand

2.13 $$\begin{aligned} \text {Im}\, s_\text {R}&= -g^2\text {Im}\,\left( \varPi ^{(-)}(s_\text {R})\right) \, , \nonumber \\ \text {Re}\, s_\text {R}&= m^2-g^2 \text {Re}\,\left( \varPi ^{(-)}(s_\text {R})\right) \, , \end{aligned}$$

where the superindex $(-)$ indicates that the pole location of a resonance is on the unphysical sheet that is defined by $\text {Im}\, q <0$. However, by imposing the conditions of Eq. (2.13) the scattering amplitude of Eq. (2.12) is fixed completely. In particular we then find for the effective coupling (setting for simplicity $H(s_p)$ from Eq. (2.4) to 1 for the *S*-wave case discussed here)

2.14 $$\begin{aligned} \tilde{g}^2 = Zg^2\, ,\qquad Z=\bigg (1+g^2\frac{\text {d}\varPi ^{(-)}(s)}{\text {d}s}\bigg |_{s=s_\text {R}}\bigg )^{-1} \, . \end{aligned}$$

In some cases this already allows for a fair representation of the pole parameters; in fact, the *P*-wave version of Eq. (2.12) closely resembles the venerable Gounaris–Sakurai parameterization for the $\rho (770)$ [3]. However, Eq. (2.12) does not have sufficient flexibility to fix pole location and residue independently, which becomes problematic for a precision description of the $\rho (770)$, and, as we will demonstrate below, it fails badly for the scalar–isoscalar $\pi \pi$ *S*-wave.

### Two-potential formalism

The goal of this work is to find a more general expression for the resonance propagator that is consistent with the fundamental field theoretic principles of unitarity, analyticity, and positivity of the spectral function of the full propagator. To reach this goal we employ the two-potential formalism [1]. It allows one to decompose the full scattering amplitude as

2.15 $$\begin{aligned} \mathcal {M}(s) = \mathcal {M}_\text {B}(s) + \mathcal {M}_\text {R}(s) \, , \end{aligned}$$

where $\mathcal {M}_\text {B}(s)$ denotes some properly chosen background amplitude. For example, in Refs. [10, 37] $\mathcal {M}_\text {B}(s)$ was chosen in such a way that the full scattering amplitude at low energies reproduced the high-precision $\pi \pi$ phase shifts from Refs. [4–6], and similarly for $\pi K$ scattering in Ref. [38]. In this way it is possible to import pertinent information on the LHCs into the resonance formalism. On the other hand, it does not allow for a straightforward evaluation of the amplitude at the resonance pole, since a continuation to the second sheet calls for an analytic continuation of the input scattering amplitude $\mathcal {M}_\text {B}$, which is not known in this case, cf. Eq. (2.19) below. Therefore, we here employ some explicit representation of the background term that allows us to perform the mentioned analytic continuation.

Since the full scattering amplitude respects the unitarity relation and so does $\mathcal {M}_\text {B}$, this does not hold for $\mathcal {M}_\text {R}$ by itself. In particular one finds

2.16 $$\begin{aligned} \mathcal {M}_\text {R}(s)&= - \frac{g^2\gamma ^2(s)}{s-m^2+g^2\varSigma (s)}\nonumber \\ {}&\equiv - \gamma (s)g\, G_\text {R}(s)\, g\gamma (s) \end{aligned}$$

for the resonance part of the scattering amplitude, with the self energy $\varSigma (s)$ now dressed by the vertex function $\gamma (s)$ to be constructed below, and

2.17 $$\begin{aligned} \mathcal {A}_\text {R}(s)= -\gamma (s)g \, G_\text {R}(s) \, \alpha \end{aligned}$$

for the production amplitude (up to a multiplicative polynomial) that originates from the resonance, with $\alpha$ quantifying the resonance–source coupling. Equation (2.16) defines the physical resonance propagator $G_\text {R}(s)$. On the physical axis the vertex function $\gamma (s)$ and the dressed self energy $\varSigma (s)$ are now linked to the background amplitude via

2.18 $$\begin{aligned} \text {disc}\,\gamma (s)&= 2i\rho (s) \mathcal {M}_\text {B}(s)^*\gamma (s) \, , \nonumber \\ \text {disc}\,\varSigma (s)&= 2i\rho (s)\left| \gamma (s)\right| ^2 \, . \end{aligned}$$

In this way the particle pairs propagating from the vertex or within the loop are not moving freely (as they do in Eq. (2.10)), but undergo interactions driven by $\mathcal {M}_\text {B}(s)$. Equation (2.18) at the same time provides a prescription for the analytic continuation of both vertex function and self energy into the unphysical sheet of the complex *s* plane, via

2.19 $$\begin{aligned} \gamma ^{(-)}(s)&= \gamma (s)\left( 1 - 2i\rho (s) \mathcal {M}_\text {B}^{(-)}(s)\right) \, , \nonumber \\ \varSigma ^{(-)}(s)&= \varSigma (s) - 2i\rho (s)\gamma ^{(-)}(s)\gamma (s) \, , \end{aligned}$$

where we need to use $\rho (s^*)=-\rho (s)^*$ for the analytic continuation of the phase-space factor from the upper to the lower half of the complex *s* plane [14].

### Explicit parameterizations

To allow for an analytic continuation of $\mathcal {M}_\text {B}$ needed in Eq. (2.19), we employ an explicit parameterization:

2.20 $$\begin{aligned} \mathcal {M}_\text {B}(s)=\frac{f_0}{f(s)-f_0\varPi (s)} \equiv \frac{1}{\rho (s)}\sin \delta _\text {B}(s) e^{i\delta _\text {B}(s)} \, , \end{aligned}$$

where the background phase $\delta _\textrm{B}$ in the expression on the far right is defined for real values of *s* above the scattering threshold only. For $\mathcal {M}_\text {B}\equiv 0$ (achieved by $f_0\rightarrow 0$) we recover the simple scattering amplitude provided in Eq. (2.12). In the general case, however, the parameter $f_0$ and the function *f*(*s*) allow us to vary both strength and phase of the residue independently of the pole location. Moreover, we can even effectively include LHCs into $\mathcal {M}_\text {B}$ by employing a polynomial in a properly chosen conformal variable $\omega (s)$ [39–41]:

2.21 $$\begin{aligned} f(s)=1+\sum _{k=1}^{k_\textrm{max}} f_k \omega ^k(s) + f_R s \, . \end{aligned}$$

The parameter $f_R$ is introduced to ensure that $\lim _{s\rightarrow \infty }\mathcal {M}_\text {B}(s)=0$, such that $\mathcal {M}_\text {B}$ and with it also $\mathcal {M}_\text {R}$ drop as 1/*s* for large values of *s*. It is not employed in the fit to the residues but is kept fixed at some sufficiently small value to keep its effect small in the resonance region. For example, in the study of the $\rho (770)$ and $f_0(500)$ presented below, we use $f_R = 1/(2\,\text {GeV})^2$, including the variation to $f_R=1/(3\,\text {GeV})^2$ in the final uncertainty estimates. It should be stressed that there is no guarantee that the given parameterization for *f*(*s*) does not lead to unphysical poles, so that checking for their absence is to be part of the analysis. For $\omega (s)$ we use the prescription [41]

2.22 $$\begin{aligned} \omega (s) = \frac{\sqrt{s-s_L}-\sqrt{s_E-s_L}}{\sqrt{s-s_L}+\sqrt{s_E-s_L}} \, , \end{aligned}$$

where $s_L$ denotes the location of the closest branch point of the LHC—for $\pi \pi$ scattering one has $s_L=0$—and $s_E$ some conveniently chosen expansion point; we use $s_E=\text {Re}\, s_\text {R}$. In the case of $\pi \pi$ scattering the leading LHC arises from two-pion exchange in the *t*- and *u*-channel, whose partial wave projection for both $\pi \pi$ *S*- and *P*-waves leads to an onset of the LHC scaling as $(-s)^{3/2}$ near $s=0$; see Appendix C. To implement this property, instead of using the parameter $\omega$ directly in Eq. (2.21) we expand in $2\omega (s)+[\omega (s)]^2$. Given this parameterization, the analytic continuation of $\mathcal {M}_\text {B}$ to the unphysical sheet simply goes by replacing $\varPi (s)$ by $\varPi ^{(-)}(s)$ in Eq. (2.20), where the latter is given by the analog of Eq. (2.19) in the absence of a background term, $\gamma (s)\rightarrow 1$.

While $\mathcal {M}_\text {B}$ is allowed to have LHCs, this is not the case for $\mathcal {M}_\text {R}$, defined in Eq. (2.16), and the production amplitude $\mathcal {A}_\text {R}(s)$, defined in Eq. (2.17). This property is guaranteed by constructing the vertex function $\gamma (s)$ from the dispersion integral

2.23 $$\begin{aligned} \gamma (s)=\exp \left( \frac{s}{\pi }\int _{s_{\text {thr}}}^\infty \frac{\text {d}s'}{s'}\frac{\delta _\text {B}(s')}{s'-s}\right) \, , \end{aligned}$$

which is the usual once-subtracted Omnès function [42]. The corresponding subtraction constant is absorbed into the coupling *g*. It is consistent with the discontinuity Eq. (2.18) and has only the right-hand cut. At the same time the information on the LHC is imported into $\mathcal {M}_\text {R}(s)$ as well as $\mathcal {A}_\text {R}(s)$ via $\delta _\text {B}(s)$. Analogously, we employ

2.24 $$\begin{aligned} \varSigma (s) = \frac{s-s_0}{\pi }\int _{s_\text {thr}}^\infty \frac{\text {d}s'}{s'-s_0}\frac{\rho (s')|\gamma (s')|^2}{s'-s} \end{aligned}$$

as a straightforward generalization of Eq. (2.10) in the presence of a background interaction. In the applications below we choose $s_0=0$. With these definitions, the scattering amplitude $\mathcal {M}$, defined in Eq. (2.15), satisfies the unitarity relation. It is important to note that from the dressed propagator, defined in Eq. (2.16), one can infer a spectral function in the standard way via

2.25 $$\begin{aligned} \sigma _\text {R}(s) = -\frac{1}{\pi }\text {Im}\, G_\text {R}(s) \, , \end{aligned}$$

which is automatically normalized

2.26 $$\begin{aligned} \int _{s_{\text {thr}}}^\infty \text {d}s \ \sigma _\text {R}(s) = 1 \, . \end{aligned}$$

This normalization condition is violated when the *s*-dependence of the real part of $\varSigma (s)$ that comes from the dispersion integral of Eq. (2.24) is abandoned.

**Table 1:** Parameters determined in the different analyses for the $\rho (770)$ as well as the resulting values for the residues. Note that the pole location is reproduced exactly by construction; cf. Eq. (4.5). The uncertainties of the bare parameters reflect the impact of the uncertainties in the input parameters, for $s_B=1/f_R=\varLambda ^2$, $\varLambda =2\,\text {GeV}$ (upper) and $\varLambda =3\,\text {GeV}$ (lower). For phase and modulus of the couplings, the first uncertainty refers to the impact of the uncertainties of the input parameters, the second one to the variation for $\varLambda \in [2,3]\,\text {GeV}$ (in scenarios (*iii*) and (*iv*), $\tilde{g}_{\rho \pi \pi }$ is reproduced exactly, by construction). Values marked with an asterisk are kept fixed in the fit

	*g*	$m \ \text{[GeV] }$	$f_0$ [GeV$^{\phantom{a}{-2}}$]	$f_1$	$|\tilde{g}_{\rho \pi \pi }|$	$\text{ arg }(\tilde{g}_{\rho \pi \pi }) \ [^\circ ]$	$|\tilde{g}_{\rho \bar{K}K}|$	$\text{ arg }(\tilde{g}_{\rho \bar{K}K}) \ [^\circ ]$
(*i*)	6.61(2)	0.84(5)	$0^*$	$0^*$	5.95(6)(1)	$-5.9(1.0)(0.7)$		
	6.66(1)	0.86(1)	$0^*$	$0^*$				
(*ii*)	6.5(1)	0.85(0)	$5(5)\times 10^{-6}$	$0^*$	5.98(5)(1)	$-5.3(9)(1)$		
	6.4(1)	0.87(1)	$10(5)\times 10^{-6}$	$0^*$				
(*iii*)	5.7(3)	0.85(2)	$2.9(1.0)\times 10^{-5}$	$-1.6(4)$	6.01(3)	$-5.3(1.0)$		
	5.8(6)	0.91(6)	$3.0(1.6)\times 10^{-5}$	$-1.0(4)$				
(*iv*)	5.9(5)	0.84(4)	$2.6(1.1)\times 10^{-5}$	$-1.6(5)$	6.01(4)	$-5.3(9)$	3.3(3)(4)	$-8.0(8)(8)$
	6.1(1.3)	0.94(15)	$3.3(1.6)\times 10^{-5}$	$-1.1(4)$				

## Generalization to higher partial waves

To extend the parameterization outlined above to partial waves with $\ell >0$, centrifugal barrier factors that grow as $q^\ell = (s/4-M^2)^{\ell /2}$ for small *s* need to be included. However, as is demonstrated, e.g., in Ref. [43], to be consistent with the positivity requirements of field theory, the physical propagator of a state is not allowed to drop faster than 1/*s* for large values of *s*. Accordingly, Eq. (2.16) tells us that the self energy $\varSigma (s)$ is not allowed to grow faster than *s* for all values of $\ell$. Thus, the energy dependence of the centrifugal barrier factors needs to be tamed. Following Ref. [44] we introduce the functions

3.1 $$\begin{aligned} \xi _\ell (s) = \sqrt{\frac{(s-4M^2)^{\, \ell }}{{2\ell +1}}} B_\ell \bigg (\frac{s-4M^2}{s_{\text {B}}-4M^2}\bigg ) \, , \end{aligned}$$

with the leading $B_\ell (x)$ given by

3.2 $$\begin{aligned} B_0=1\, , \qquad B_1(x)=\sqrt{1/(1+x)} \, . \end{aligned}$$

Explicit forms for barrier factors with values of $\ell$ up to 4 are given, e.g., in Ref. [45]. Here $s_\text {B}$ denotes some properly chosen scale with $s_\text {B}>4M^2$. The final results should not depend strongly on this parameter; for definiteness we choose $s_\text {B}=1/f_R=\varLambda ^2$, $\varLambda =2\,\text {GeV}$, in the analyses below. This is the scale that gives the best results for the naive resonance model without a background interaction, see row (*i*) of Table 1, but we again include the variation to $\varLambda =3\,\text {GeV}$ in the final uncertainty estimates. These regulator functions introduce unphysical singularities for space-like values of *s*. However, since they are far away for the given choice of parameters and pushed to the unphysical sheet by construction in both the vertex functions and the self energies, they have no significant effect on the resonance parameters and line shapes.

In this work we restrict ourselves to systems of two spinless particles such that the total angular momentum is captured in $\ell$. Then we can adapt the expressions from above to the case $\ell \ne 0$ by employing

3.3 $$\begin{aligned} \varSigma _\ell (s) = \frac{s-s_0}{\pi }\int _{s_\text {thr}}^\infty \frac{\text {d}s'}{s'-s_0}\frac{\rho (s')\xi _\ell (s')^2|\gamma _\ell (s')|^2}{s'-s} \, . \end{aligned}$$

The expression for $\varPi _\ell (s)$ follows from the one above by putting $\gamma _\ell (s)$ to 1 and choosing $s_0=4M^2$. The vertex functions $\gamma _\ell (s)$ are still evaluated from Eq. (2.23); however, they need to be constructed from the phase of the adapted $\mathcal {M}_\text {B}$, which now reads

3.4 $$\begin{aligned} \mathcal {M}_\text {B}=\frac{\xi _\ell (s)^2f_0}{f(s)-f_0\varPi _\ell (s)} \, . \end{aligned}$$

For the resonance amplitude and the production amplitude we find

3.5 $$\begin{aligned} \mathcal {M}_\text {R}(s)&= - g^2\gamma _\ell (s)^2\xi _\ell (s)^2 \, G_\text {R}(s) \nonumber \\&= -\frac{g^2\gamma _\ell (s)^2\xi _\ell (s)^2}{s-m^2+g^2\varSigma _\ell (s)} \, , \nonumber \\ \mathcal {A}_\text {R}(s)&= - g \gamma _\ell (s)\xi _\ell (s) \, G_\text {R}(s) \, \alpha \, . \end{aligned}$$

With this definition for the resonance propagator $G_\text {R}(s)$, the spectral function introduced in Eq. (2.25) remains normalized according to Eq. (2.26) for all values of $\ell$. In contrast, using the expression for $G_\text {R}(s)$ provided in Ref. [3], the resulting spectral function is not normalized due to the missing barrier factors $B_\ell (s)$, leading to a resonance propagator that drops as $1/(s\log (s))$ for large values of *s*. One key advantage of our formalism is that the resulting spectral function is automatically normalized, which is not the case when improving Breit–Wigner-type parameterizations of the imaginary part of a resonance propagator via a dispersion integral [46–49]. In this sense, we obtain a more direct implementation of the corresponding Källén–Lehmann spectral representation [50, 51] for a given resonance.

## Application to the $\rho (770)$

Before generalizing the formalism to coupled channels, we illustrate its application to the $\rho (770)$ and $f_0(500)$ resonances, respectively. Pole parameters with very high accuracy are available, e.g., from Refs. [16, 52]:

4.1 $$\begin{aligned} M_\rho&= 762.5(1.7) \,\text {MeV}\, , \nonumber \\ \varGamma _\rho&= 2\times 73.2(1.1) \,\text {MeV}\, , \nonumber \\ \tilde{g}_{\rho \pi \pi }&= 6.01(8)\exp {\left\{ - i \frac{\pi }{180}5.3(1.0)\right\} } \, . \end{aligned}$$

**Fig. 1:**
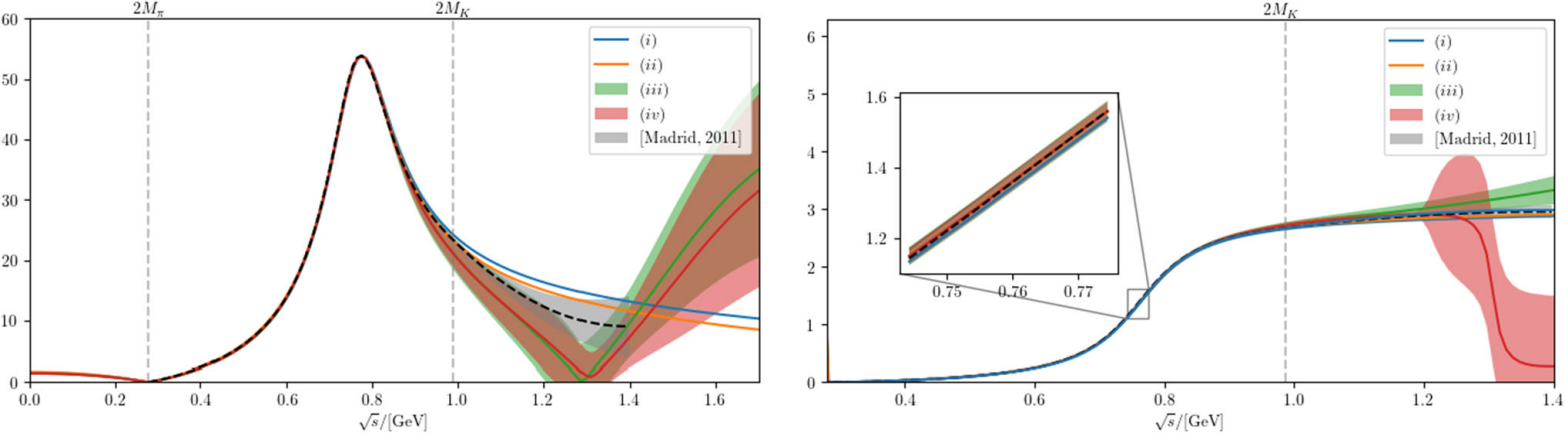
The left (right) figure shows the absolute value (phase) of the $\pi \pi$ scattering amplitude, in both cases for the various analyses presented here: (*i*), (*ii*), (*iii*), and (*iv*) are shown as the blue, orange, green, and red line or band, respectively. In both figures we also show for comparison the results from Ref. [6]

**Fig. 2:**
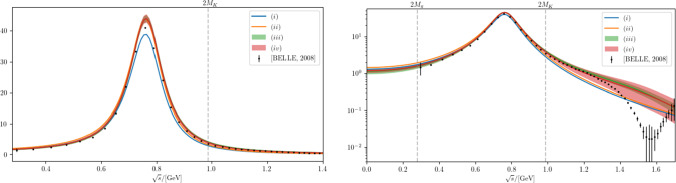
The pion vector form factor compared to data derived from $\tau ^-\rightarrow \pi ^-\pi ^0\bar{\nu }_\tau$ [53] on a linear scale (left) and on a logarithmic scale (right). Legend as in Fig. 1

The vector form factor is defined via the current matrix element

4.2 $$\begin{aligned} {\langle {\pi ^+(p_1)\pi ^-(p_2)}|}j_{\mu }^{(I=1)}{|{0}\rangle }=(p_1-p_2)_{\mu }F_\pi ^V(s)\, , \end{aligned}$$

where $j_\mu ^{(I=1)} = (\bar{u}\gamma _\mu u-\bar{d}\gamma _\mu d)/2$ and $s=(p_1+p_2)^2$. In the formalism introduced above it takes the form

4.3 $$\begin{aligned} F_\pi ^V(s)=B_{1}\bigg (\frac{s-4M_\pi ^2}{s_B-4M_\pi ^2}\bigg )\frac{-\alpha g \gamma _1(s)}{s-m^2+g^2\varSigma _1(s)}\, , \end{aligned}$$

with the barrier factor $B_1(x)$ defined in Eq. (3.2). This allows us to determine $\alpha$ in Eq. (2.17) via the coupling of the $\rho (770)$ to the photon

4.4 $$\begin{aligned} \tilde{g}_{\rho \gamma }=5.01(7)\exp \left\{ -i\frac{\pi }{180}1(1)\right\} \, \end{aligned}$$

as provided in Ref. [52]. We emphasize that Eq. (4.3) does not yet define a suitable parameterization for precision studies of the pion vector form factor, for the following reasons: first, $F_\pi ^V(s)$ is not normalized exactly to $F_\pi ^V(0)=1$, since we only included the pole position and residues of the $\rho (770)$ as constraints, and this minimal parameterization violates the normalization by about $5\%$. Second, the barrier factor $B_1$ ensures a normalized spectral function, but introduces an unphysical LHC starting at $s=-(s_B-8M_\pi ^2)$. Accordingly, the dispersion relation for $\text {Re}\, F_\pi ^V(s)$ in the physical region around the $\rho (770)$ is violated by (2–$3)\%$, a reasonably small effect given the scale $s_B\gtrsim 4\,\text {GeV}^2$. Third, $F_\pi ^V(s)$ behaves asymptotically as $1/s^{3/2}$, in contradiction to the expected 1/*s* scaling [54–58]. These shortcomings can be remedied by extending Eq. (4.3) appropriately, using the freedom in the choice of barrier factors and taking into account polynomial terms in the unitarity relation for $F_\pi ^V(s)$. Such generalizations will be studied in future work, while here we show the results for the minimal form (4.3).

To demonstrate the effect of the background amplitude on the properties and line shape of the $\rho (770)$ we applied three variants thereof: (*i*) $\mathcal {M}_\text {B}\equiv 0$, (*ii*) $k_\textrm{max} = 0$, and (*iii*) $k_\textrm{max} = 1$, where the parameter $k_\textrm{max}$, introduced in Eq. (2.21), counts the number of terms in the expansion in the conformal variable.

Since the parameters of the background amplitude $\mathcal{M}_\textrm{B}$ enter the expression for the resonance amplitude through an integral that can only be performed numerically, it is not possible to calculate the residue and its phase directly from the model parameters. We therefore fit its parameters to the residue, while at all times demanding

4.5 $$\begin{aligned} \text {Im}\, s_\text {R}&= -g^2\text {Im}\,\left( \varSigma _\ell ^{(-)}(s_\text {R})\right) \, , \nonumber \\ \text {Re}\, s_\text {R}&= m^2-g^2 \text {Re}\,\left( \varSigma _\ell ^{(-)}(s_\text {R})\right) \, , \end{aligned}$$

where in case of the $\rho (770)$ discussed in this section we have $\ell =1$. In this way the correct pole location is guaranteed. The results of the three different analyses are shown in Table 1. The uncertainties quoted in the table were determined via a bootstrap method, where both residue and pole location were varied within their allowed uncertainties in the course of the analysis—always demanding that there be no additional singularities appearing in the amplitude. It is the latter condition that leads to a slightly smaller uncertainty in the deduced residues than in the input residue. This limitation could be overcome by allowing for more parameters in the conformal expansion, to extend the region that can be scanned in the bootstrap procedure, but we restrict the analysis to the minimal case in which all parameters can be determined directly from the residues.

**Table 2:** Parameters determined in the different analyses for the $f_0(500)$ as well as the resulting values for the residues. Note that the pole location is reproduced exactly by construction; cf. Eq. (4.5). The uncertainties of the bare parameters reflect the impact of the uncertainties in the input parameters, for $f_R=1/\varLambda ^2$, $\varLambda =2\,\text {GeV}$ (upper) and $\varLambda =3\,\text {GeV}$ (lower). For phase and modulus of the couplings, the first uncertainty refers to the input parameters, the second one (where applicable) to the variation for $\varLambda \in [2,3]\,\text {GeV}$. Values marked with an asterisk are kept fixed in the fit

	*g*	*m*	$$f_0$$	$$f_1 $$	$$g_{\sigma \bar{K}K}$$	$$|\tilde{g}_{\sigma \pi \pi }|$$	$$\text{ arg }(\tilde{g}_{\sigma \pi \pi }) \ [^\circ ]$$	$$|\tilde{g}_{\sigma \bar{K}K}|$$	$$\text{ arg }(\tilde{g}_{\sigma \bar{K}K}) \ [^\circ ]$$
	$\text{[GeV] }$	$\text{[GeV] }$			$\text{[GeV] }$	$\text{[GeV] }$		$\text{[GeV] }$	
(*i*)	3.0(1)	0.14(3)	$0^*$	$0^*$	$0^*$	3.12(18)	10(5)		
(*ii*)	5.3(5)	0.89(6)	$-25.5(1.1)$	$0^*$	$0^*$	3.33(17)(8)	$-73.1(2.4)(0.5)$		
	7.5(2.2)	1.15(15)	$-27.5(1.3)$	$0^*$	$0^*$				
(*iii*)	4.7(3)	0.82(3)	$-24.8(6)$	0.06(2)	$0^*$	3.61(11)	$-74.0(2.2)$		
	5.3(4)	0.82(5)	$-24.8(6)$	0.06(2)	$0^*$				
(*iv*)	4.8(2)	0.80(3)	$-24.9(4)$	0.06(2)	2.3(2)	3.61(12)	$-74.0(2.3)$	2.0(1)	$-23(1)(1)$
	5.4(6)	0.85(8)	$-26.2(7)$	0.07(3)	2.4(2)				

The results show that in case of the $\rho (770)$ already the model without any background gives a reasonable prescription of the residue. This should not come as a surprise, given that the resulting amplitudes are very close to the Gounaris–Sakurai parameterization [3], with the only difference that we employ the barrier function $B_\ell$, which, however, has a minor impact in the resonance region. As soon as we allow for a background amplitude, both the absolute value and phase of the residue can be exactly reproduced. The resulting $\pi \pi$ *P*-wave scattering amplitude and the pion vector form factor are shown in Figs. 1 and 2, respectively.

Two features of our analysis are worth noting. First, the energy dependence of both the scattering amplitude and the form factor are reproduced with rather high accuracy in all analyses, just using the correct $\rho$ pole parameters as well as the correct analytic structure of the amplitudes. Second, as becomes evident in the right panel of Fig. 2, in the analysis (*iii*), which reproduces the central values of the $\rho$ pole parameters exactly, the background amplitude introduces some resonance-like structure right in the mass range of the $\rho '$, which might indicate that the deviation of the $\rho$ pole parameters from the most naive implementation of the resonance physics realized in analysis (*i*) is driven by the excited vector states.

## Application to the $f_0(500)$

**Fig. 3:**
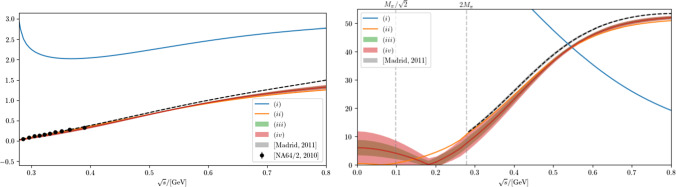
Comparison of the phase shifts (left) and the absolute value of the scattering amplitude (right) that result for the scalar–isoscalar $\pi \pi$ channel, once the pole parameters are fixed via the different variants of the model: (*i*), (*ii*), (*iii*), and (*iv*) are shown as the blue, orange, green, and red line or band, respectively. The black dashed line shows the phase shift and the related absolute value of the scattering amplitude (between $\pi \pi$ and $\bar{K}K$ threshold) from Ref. [4] for comparison. The dots show the phase shifts extracted from $K_{e4}$ decays [59]. The first and second perpendicular lines show the locations of the Adler zero and the $\pi \pi$ threshold, respectively

For the scalar–isoscalar channel, we use the pole parameters [16, 52]

5.1 $$\begin{aligned} M_\sigma&= 458(14) \,\text {MeV}\, , \nonumber \\ \varGamma _\sigma&=\ 2\times 261(10) \,\text {MeV}\, , \nonumber \\ \tilde{g}_{\sigma \pi \pi }&= 3.61(13)\exp {\left\{ -i \frac{\pi }{180}74(3)\right\} } \,\text {GeV}\, . \end{aligned}$$

The scalar form factor is defined via the current matrix element

5.2 $$\begin{aligned} {\langle {\pi \pi }|}j_{S}{|{0}\rangle }=M_{\pi }^2 F_\pi ^S(s)\,, \end{aligned}$$

with the scalar current $j_{S}=\hat{m}(\bar{u}u+\bar{d}d)$. In the formalism outlined above it takes the form

5.3 $$\begin{aligned} F_\pi ^S(s)=\frac{-\alpha g \gamma (s)}{s-m^2+g^2\varSigma (s)}\, . \end{aligned}$$

This allows us to determine $\alpha$ in Eq. (5.3) via the coupling of the $f_0(500)$ to a scalar source

5.4 $$\begin{aligned} \tilde{g}_{\sigma S}=151(5)\exp {\left\{ -i \frac{\pi }{180}25(2)\right\} }\,\text {MeV}\end{aligned}$$

as given in Ref. [52], see Appendix A.

**Fig. 4:**
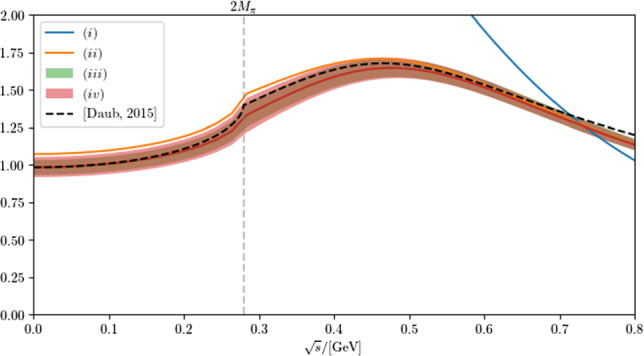
Two-pion production amplitude that results for the different fits. The color code agrees with that of Fig. 3, only that now the dashed line is the non-strange scalar pion form factor of Ref. [21], derived from the phase shifts of Ref. [4]

As in the case of the $\rho (770)$, we perform three different analyses, with different levels of sophistication for the background amplitude. As before, in all cases the pole locations are reproduced exactly by employing the *S*-wave version of Eq. (4.5). The results are reported in Table 2. As one can see, in the absence of a background the residue of the $f_0(500)$ is not well described; in particular, the phase of the residue is off completely. Also the resulting phase shifts and amplitudes have little in common with our empirical knowledge of the scalar–isoscalar $\pi \pi$ amplitude, cf. the blue curves in Figs. 3 and 4. In this case the resonance amplitude acquires an additional pole right below threshold on the first sheet, in contradiction to the physical $\pi \pi$ scattering amplitude. These observations reflect the fact that the features of the $f_0(500)$ cannot be captured by a Breit–Wigner function, even if an energy-dependent width is included. The situation improves drastically when we allow for the simplest background amplitude, and especially as soon as the LHC is included in the parameterization the residue is reproduced exactly, in line with our modern understanding of the physics of the $f_0(500)$ resonance [14, 60]. With a non-vanishing background included in the analysis, fit (*ii*), phase and absolute value of the residue are improved significantly. At the same time the unphysical pole disappears and is replaced by a zero in the amplitude right below threshold, see the orange lines in Figs. 3 and 4. When we include the $f_1$-term, the phase and absolute value of the residue are reproduced exactly. Also for this parameterization we find a zero in the amplitude in the same energy range. This is illustrated by the green curves in Figs. 3 and 4.

The features described above demonstrate the intimate relation between the properties of the $f_0(500)$ and a non-trivial energy dependence of the $\pi \pi$ scattering amplitude in the threshold region. In fact, since pions are the Goldstone bosons of the spontaneously broken chiral symmetry of QCD, there is necessarily a zero just below threshold in the *S*-wave isoscalar $\pi \pi$ scattering amplitude, the Adler zero [22, 23], which at leading order (LO) in ChPT is located at $s_A=M_\pi ^2/2$; as we show in Appendix D, this prediction is remarkably stable towards high-order corrections, with the one- and two-loop contributions reducing the LO value by $12\%$ and $3\%$, respectively. Since we derive the amplitudes from the pole parameters only, it should not come as a surprise that the threshold physics driven by the chiral properties of QCD is not exactly reproduced, however, it is a remarkable observation that reproducing the pole properties of the $f_0(500)$ with an amplitude consistent with unitarity and analyticity seems to be possible only with amplitudes that feature a zero in the scattering amplitude just below threshold, which finds a natural explanation in the approximate chiral symmetry of QCD. The zeros found in the amplitudes are $s_A^{(ii)}=0.09\,M_{\pi }^{2}$, $s_A^{(iii)}=2.1(4)\,M_{\pi }^{2}$, and $s_A^{(iv)}=2.1(1.1)\,M_{\pi }^{2}$. While it has been known for a long time that unitarizing an amplitude that is in line with chiral constraints leads to a pole of the *S*-matrix in close proximity to that of the $f_0(500)$ [24, 25], what we have demonstrated here for the first time is the opposite direction.

Finally, the black dashed lines in the two panels of Figs. 3 and 4 show the correct phase shifts, absolute value of the scattering amplitude, and production amplitude, respectively. They are based on the high-precision phase shifts of Ref. [4]. The plots clearly illustrate that as soon as we include the background interaction, the qualitative features of the $\pi \pi$ amplitude are reproduced reasonably well.

## Generalization to coupled channels and branching fractions

### Coupled channels

For a meaningful discussion of branching fractions we need to extend the formalism introduced above to multiple channels, whose number shall be denoted by $n_c$. Since the goal of this study is to deduce line shapes from resonance properties and the residues factorize, it appears justified to introduce the background amplitude in diagonal form; however, the generalization to a non-diagonal background is straightforward [37]. Thus we write

6.1 $$\begin{aligned} \mathcal {M}_\text {B}(s)_{ad} = \delta _{ad}\frac{\xi _{a}^2(s)f_{0\, a}}{f_a(s)-f_{0\, a}\varPi _a(s)} \, , \end{aligned}$$

where $a,d\in \{1,\ldots ,n_c\}$, $\varPi _a(s)$ denotes the non-interacting renormalized self energy of channel *a*, and $\xi _{a}(s)$ is the corresponding, channel-specific centrifugal barrier factor. In the multi-channel case the channel label not only specifies the particle content of the given channel, but also the angular momentum. Then the expression for the physical propagator reads

6.2 $$\begin{aligned} G_\text {R}(s)= \bigg (s-m^2+\sum _{a=1}^{n_c} g_a^2\varSigma _a(s)\bigg )^{-1} \, , \end{aligned}$$

and we obtain

6.3 $$\begin{aligned} \mathcal {M}_R(s)_{ad}&= - \xi _a(s)\gamma _a(s) g_a \, G_\text {R}(s) \, g_d \gamma _d(s) \xi _d(s) \, , \nonumber \\ \mathcal {A}_\text {R}(s)_a&= - g_a \gamma _a(s)\xi _a(s) \, G_\text {R}(s) \, \alpha \, , \end{aligned}$$

where $\gamma _a(s)$ is the vertex function that emerges from the background amplitude $\mathcal {M}_\text {B}(s)_{aa}$ according to Eqs. (2.20) and (2.23). If we allowed for off-diagonal terms in $\mathcal {M}_\text {B}(s)_{ad}$, also the self energies would acquire off-diagonal terms [37].

For $n_c$ coupled channels one is faced with $2^{n_c}$ Riemann sheets. The resonance propagator $G_\text {R}(s)$ on some arbitrary sheet can be written as

6.4 $$\begin{aligned} G_\text {R}^{(\text {sh}_1,\ldots ,\text {sh}_{n_c})}(s) = \Bigg (s-m^2+\sum _{a=1}^{n_c} g_a^2\varSigma _a^{(\text {sh}_a)}(s)\Bigg )^{-1} \, , \end{aligned}$$

where the index $\text {sh}_a\in \{+,-\}$ specifies on which sheet with respect to channel *a* the self-energy function needs to be evaluated. In the single-channel analysis, the real and imaginary parts of the pole location allowed us to determine *m* and $g^2$ for any given background; see Eq. (4.5). The situation is a little more complicated now, since various channels and the corresponding couplings appear in the denominator of the resonance propagator $G_\text {R}(s)$ defined in Eq. (6.2). In practice, the procedure to fix the proper pole location(s) depends on what information is available for the resonance under study. For example, if pole locations on various sheets are known, one may straightforwardly generalize Eq. (4.5) to fix $g_a$ as well as *m*. If, however, only one pole is known, then Eq. (4.5) only determines one of the $g_a$ couplings and the others may be employed to fix the pertinent residues. In the examples discussed in this paper, the two-pion channels are by far dominating, and we therefore use Eq. (4.5) as given to fix *m* and $g_{\pi \pi }$. The additional inclusion of the couplings to relatively unimportant inelastic channels, like $\gamma \gamma$ for the $f_0(500)$, only changes the pole location within uncertainties. Therefore, we will use for those the approximation

6.5 $$\begin{aligned} g^2_{a}=|\tilde{g}_{a}|^2/|Z| \end{aligned}$$

to find the corresponding couplings. For more strongly coupled channels, such as the $\bar{K} K$ channel in the scalar–isoscalar $\pi \pi$ system, we implement the bare coupling as an additional free parameter in the fit to reproduce the residues.

### Branching fractions

In this subsection we compare different possible definitions for branching fractions and propose a new one based on the formalism discussed in the previous sections.

In Eq. (1.1) the total width of a resonance was fixed from the pole location $s_\text {R}$ as $\varGamma _\text {R}= -\text {Im}\, s_\text {R}/M_\text {R}$. Matching that to Eq. (6.4) reveals a natural definition of partial widths, namely

6.6 $$\begin{aligned} \varGamma _a^{\text {R}\mathrm (p)} = -g^2_a\text {Im}\,(\varSigma _a^{(\text {sh}_a)}(s_\text {R}))/M_\text {R}\, , \end{aligned}$$

where the index $(\text {sh}_a)$ is fixed by the sheet on which the pole at $s_\text {R}$ is located. Clearly such a definition is sensible only if it is just a single pole that dominates the physics, however, this holds true both for the $f_0(500)$ and the $\rho (770)$. In the absence of background interactions for a single channel, this definition agrees with the one of Ref. [61]. For the evaluation of the branching ratio from the pole location, one then obtains

6.7 $$\begin{aligned} \text {Br}_a^\mathrm{(p)} = \varGamma _a^{\text {R}\mathrm (p)}/\varGamma _\text {R}\, . \end{aligned}$$

However, it was shown in Ref. [26] using the example of $f_0(980)$ and $a_0(980)$ (and further discussed for the former resonance in Ref. [28]) that this definition runs into problems when the most relevant pole sits on a sheet other than the one adjoined to the physical sheet above all thresholds. Taking the $f_0(980)$ as an example, where the pole typically sits above the $\bar{K}K$ threshold, but on the physical sheet with respect to the $\bar{K}K$ channel, the problem is that the different contributions to the imaginary part of the pole location in Eq. (6.2) no longer add up, since $\text {Im}\,(\varSigma _{f_0(980)\bar{K}K}^{(+)}(s_{f_0(980)}))>0$ but $\text {Im}\,(\varSigma _{f_0(980)\pi \pi }^{(-)}(s_{f_0(980)}))<0$. The authors argue that in this case $\varGamma _\text {R}$ is not a proper measure of the total width. Adapting their insights to our parameterization, we define a modified expression from the one given by the pole location:

6.8 $$\begin{aligned} \varGamma _a^{\text {R}\mathrm (p,m)}&= g^2_a\left| \text {Im}\,(\varSigma _a^{(\text {sh}_a)}(s_\text {R}))/M_\text {R}\right| \, , \nonumber \\ \varGamma _\textrm{tot}^\text {R}&= \sum _a \varGamma _a^{\text {R}\mathrm (p,m)} \, , \nonumber \\ \text {Br}_a^\mathrm{(p,m)}&= \varGamma _a^{\text {R}\mathrm (p,m)}/\varGamma _\textrm{tot}^\text {R}\, . \end{aligned}$$

It should be stressed that the mentioned sign problem highlighted for the example of the $f_0(980)$ is considerably more general: it occurs as soon as channels are included in the analysis that are closed at the resonance location, as is the case for the decay of both $f_0(500)$ and $\rho (770)$ to two kaons. Thus, we also include $\text {Br}_a^\mathrm{(p,m)}$ in our study.

In some cases, such as the two-photon coupling of the $f_0(500)$, a narrow-width formula has been used in the literature to turn the residue into a decay rate [17, 30–32]. Thus, in this prescription one has

6.9 $$\begin{aligned} \varGamma _a^{\text {R}\mathrm (nw)}=\frac{|\tilde{g}_a|^2}{M_R}\rho _a(M_R^2)\xi ^2_a(M_R^2) \, , \end{aligned}$$

where the superscript (nw) refers to the narrow-width limit. To see how well this prescription works in practice, we define

6.10 $$\begin{aligned} \text {Br}_a^\mathrm{(nw)} = \varGamma _a^{\text {R}\mathrm (nw)}/\varGamma _\text {R}\, . \end{aligned}$$

Contrary to the branching ratios defined in Eq. (6.7), those of Eq. (6.10) not necessarily add to 1. Only for narrow states above threshold, where $M_\text {R}^2-4M_a^2\gg M_\text {R}\varGamma _\text {R}$ holds, one finds

6.11 $$\begin{aligned} g^2_a\text {Im}\,(\varSigma _a^{(-)}(s_\text {R}))\approx -|\tilde{g}_a|^2 \rho _a(M_\text {R}^2) \xi ^2_a(M_R^2)\, , \end{aligned}$$

and Eq. (6.9) is recovered naturally. However, for $M_\text {R}^2 - 4M_a^2<0$ the phase-space factor $\rho _a(M_R^2)$ vanishes and thus for that case the narrow-width formula does not provide a meaningful answer.

The definition we propose to use for the evaluation of branching fractions is a lot closer to what is measured in experiment for a single, isolated resonance. What is done there can be summarized as [28]

6.12 $$\begin{aligned} \text {Br}_a^\textrm{exp} = N_a/N_\textrm{tot} \, , \qquad N_\textrm{tot} =\sum _{a=1}^{n_c} N_a \, , \end{aligned}$$

where $N_a$ is the number of events measured for the decay of the resonance R into channel *a* in some production reaction (assuming that the resonance leaves a sufficient imprint in the channel). The specifics of the production reaction cancel in the ratio and thus $\text {Br}_a^\textrm{exp}$ measures a resonance property. Given that the count rates in a channel *a* from some resonance $\text {R}$ are calculable from $G_\text {R}$, evaluated on the physical sheet such that $\text {sh}_a=+$ for all *a* (the superindex introduced in Eq. (6.4) is dropped here to ease notation), we can write

6.13 $$\begin{aligned} \text {Br}_a^\mathrm{(cr)} = \int _{s_{\text {thr}}^a}^\infty \frac{\text {d}s}{\pi } \left| G_\text {R}(s) g_a \gamma _a(s)\xi _{a}(s)\right| ^2 \rho _a(s)\, , \end{aligned}$$

where the label (cr) shows the relation to the count rates. Since the formalism described above automatically generates a spectral function that is normalized, the sum over the $\text {Br}_a^\mathrm{(cr)}$ is one. Moreover, all the self energies need to be evaluated on the physical, the $(+)$, sheet and accordingly no sign problem can appear, regardless of where the pole is located.

The construction Eq. (6.13) encodes the properties of a single resonance. As long as pole locations and residues are known with sufficient accuracy for each individual state, it should also be applicable for partial waves with various overlapping resonances, although in this case the method sketched in Eq. (6.12) can no longer be applied to experimental data straightforwardly. Concrete tests hereof, including the sensitivity to the parameterization of background amplitude and barrier factors as studied here for the $\rho (770)$ and $f_0(500)$, are left for future work.

**Table 3:** Comparison of the branching ratios calculated using the different prescriptions introduced in Sect. 6.2. The dagger indicates that for those branching fractions the uncertainties could not be evaluated, for the reasons detailed in the main text. Whenever two uncertainties are provided, the first one refers to that in the input quantities, the second one to the variation for $\varLambda \in [2,3]\,\text {GeV}$; see Tables 1 and 2

Resonance	Channel (*a*)	Narrow width	Pole location		Ref. [28]		This work
		$\text {Br}_a^\mathrm{(nw)}$	$\text {Br}_a^\mathrm{(p)}$	$\text {Br}_a^\mathrm{(p,m)}$	$\text {Br}_a^\mathrm{(B)}$	$\text {Br}_a^\mathrm{(B,n)}$	$\text {Br}_a^\mathrm{(cr)}$
$f_0(500)$	$\pi \pi$	0.8(1)	1.03(1)(0)	0.97(0)(0)	0.52(8)	0.94(5)	0.970(5)(12)
	$\gamma \gamma \ \times 10^{6}$	3.0(7)	5.0(1.6)(0.3)	4.8(1.5)(0.3)	1.9(6)	3.5(9)	1.4(4)(3)
	$\bar{K}K$	0	$-0.03(1)(0)$	0.03(1)(0)	0.03(2)	0.06(4)	0.030(5)(12)
	sum	0.8	1.0	1.0	0.74	1.0	1.0
$\rho (770)$	$\pi \pi$	1.007(14)	1.04(1)(4)	0.96(1)(3)	1.222(5)	0.967(3)	0.95(4)(3)
	$\pi \gamma \ \times 10^{4}$	5.1(1.1)	3(1)(6)	3(1)(3)	$5.4^\dagger$	$4.3^\dagger$	12(1)(4)
	$\bar{K}K$	0	$-0.05(1)(3)$	0.04(1)(3)	0.0419(5)	0.0331(3)	0.05(4)(3)
	sum	1.0	1	1	1.26	1	1

As a final definition, we compare our results to Eq. (28) in combination with Eq. (19) of Ref. [28]:

6.14 $$\begin{aligned} \text {Br}_a^\mathrm{(B)} = \int _{s_{\text {thr}}^a}^\infty \frac{\text {d}s}{\pi } \frac{f|\tilde{g}_a|^2\rho _a(s)\xi _{a}(s)^2}{\left| s-\hat{m}^2+i f\sum _b |\tilde{g}_b|^2 \rho _b(s)\xi _{b}(s)^2\right| ^2} \, , \end{aligned}$$

where as before the $\tilde{g}_a$ denote the effective couplings derived from the residues. However, as discussed in Sect. 2, without additional background contributions it is not possible to simultaneously obtain both the correct pole location and residue. Because of this, the authors of Ref. [28] introduced a fudge factor, *f*, adjusted along with $\hat{m}^2$ in such a way that the pole location is correct. Thus, Eq. (6.14) is close to what one would obtain in our formalism for a vanishing background, only that the dispersive pieces of the self energies are dropped. As discussed above, Eq. (6.14) does in general not lead to the correct residues, however, for the $\rho (770)$, this is not necessarily a big effect. Moreover, Eq. (6.14) relates to a spectral function that is not normalized, resulting in branching fractions that do not sum to 1. Thus, to allow for a better comparison, we also introduce a normalized branching ratio based on Eq. (6.14), namely

6.15 $$\begin{aligned} \text {Br}_a^\mathrm{(B,n)} = \frac{1}{N}\text {Br}_a^\mathrm{(B)}\, , \qquad N=\sum _{a=1}^{n_c}\text {Br}_a^\mathrm{(B)} \, . \end{aligned}$$

### Results for the $\rho (770)$ and $f_0(500)$

Using the examples of the $\rho (770)$ and $f_0(500)$, we now compare the results of the various prescriptions to calculate branching fractions, including the dominant $\pi \pi$ decay as in Sects. 4 and 5, but including as well the $\gamma \gamma$ and $\bar{K}K$ channels for the $f_0(500)$ [32, 33]

6.16 $$\begin{aligned} \tilde{g}_{f_0(500)\gamma \gamma }&= 6.3(7)\exp {\left\{ -i\frac{\pi }{180}115\right\} } \,\text {MeV}\, , \nonumber \\ \tilde{g}_{f_0(500)\bar{K} K}&= 2.1(4)\exp {\left\{ -i\frac{\pi }{180} 57.9\right\} } \,\text {GeV}\, , \end{aligned}$$

and the $\pi \gamma$ and $\bar{K} K$ ones for the $\rho (770)$. The former has the residue [62]

6.17 $$\begin{aligned} \tilde{g}_{\rho \pi \gamma }=\sqrt{8\pi \alpha _{\text {em}}}\ 0.79(8) \,\text {GeV}^{-1} \, , \end{aligned}$$

where $\alpha _{\text {em}}$ is the fine-structure constant, with a phase consistent with zero. Compared to the coupling of the pions to the $\rho (770)$, the dimension is different due to an additional momentum dependence in the vertex; see Appendix B for details.

The residue for the coupling of the $\rho (770)$ to $\bar{K}K$ is estimated using an SU(3) symmetric vector-meson-dominance Lagrangian [63, 64] and by comparing the vector–isovector $\pi \pi$ and $\bar{K}K$ form factors (cf. also Ref. [65]). With that we can approximate the bare coupling of the $\rho$ to the kaons as $g_{\rho \bar{K}K}^2=g_{\rho \pi \pi }^2/2$ to obtain a prediction for the value of the residue.

The branching ratios calculated for these systems with the different methods are shown in Table 3. As expected from Eq. (6.11), for those cases in which the inelastic threshold is well below the resonance mass, as is the case for the $\gamma \gamma$ decay of the $f_0(500)$ and the $\pi \gamma$ decay of the $\rho (770)$, the narrow-width formula, $\text {Br}_a^\mathrm{(nw)}$, gives results (almost) consistent with the ones derived from the pole location, $\text {Br}_a^\mathrm{(p)}$. However, some deviations are observed in comparison to $\text {Br}_a^\mathrm{(cr)}$, which reflects the impact of the line shape on the branching fractions—note that $s_\text {thr}^{\gamma \gamma }=0$ and $s_\text {thr}^{\pi \gamma }=M_{\pi }^{2}$, so that the line shape is probed over a large range when the integral in Eq. (6.13) is evaluated.

The effective prescription from Ref. [28], $\text {Br}_a^\mathrm{(B)}$, suffers from the wrong normalization of Eq. (6.14). Therefore, already the $\pi \pi$ branching ratio deviates significantly from the other cases. If one corrects for this, the agreement with $\text {Br}_a^\mathrm{(cr)}$ improves, as shown in column $\text {Br}_a^\mathrm{(B,n)}$. However, employing $\text {Br}_a^\mathrm{(B,n)}$ to calculate the two-photon width of the $f_0(500)$ gives a result that is two standard deviations larger than the reference value provided in the column marked as $\text {Br}_a^\mathrm{(cr)}$. This large discrepancy follows from the increased sensitivity to the line shape of the $f_0(500)$ at small values of *s*, which in this parameterization becomes similar to the blue solid line in Fig. 4.

We were not able to determine the uncertainties for the $\pi \gamma$ branching fraction of the $\rho (770)$ for $\text {Br}_a^\mathrm{(B)}$ and $\text {Br}_a^\mathrm{(B,n)}$. The reason is that the integrand in Eq. (6.14) develops a pole below the two-pion threshold, since the analytic continuation of the $\bar{K}K$ momentum becomes sizable here and contributes negatively. The same problem does not occur for Eq. (6.13), since here the analytic continuation of the momentum is tamed by the dispersion integral. Furthermore, in the case of $s_B = (3\,\text {GeV})^2$, we observe that the imaginary part of the $\pi \gamma$ self energy on the second sheet at the $\rho (770)$ pole location changes sign compared to the central solution at $\varLambda =2\,\text {GeV}$. Such zeros on the second sheet also occur for other channels, but the $\pi \gamma$ case is the only one for which we find a strong sensitivity of its position to the regulator scale. In contrast, the behavior on the real axis appears to be more stable, suggesting that indeed $\text {Br}^\mathrm{(cr)}_a$ defines a better prescription for a branching fraction than $\text {Br}^\mathrm{(p)}_a$.

When the threshold for the inelastic channel lies above the resonance location, as for the $\bar{K}K$ decay of the $f_0(500)$ and $\rho (770)$, the various expressions naturally give very different results, and $\text {Br}_a^\mathrm{(nw)}$ even becomes zero. However, also the prescription via the pole location that appears improved at first glance, $\text {Br}_a^\mathrm{(p)}$, gives a negative value for both resonances, and thus does not produce a meaningful branching fraction in this case either. All other prescriptions give consistent results within uncertainties. The uncertainty of the branching fraction $\text {Br}_{\rho \rightarrow \bar{K}K}^\mathrm{(cr)}$ is significantly larger than all others; this reflects the fact that the $\rho (770)$ line shape is badly determined for energies beyond $1\,\text {GeV}$, where it is probed for this channel.

## Summary and outlook

In this paper we introduced a formalism consistent with the fundamental principles of analyticity, unitarity, and positivity of the spectral function that allows one to derive line shapes of a resonance solely from its pole parameters. The resulting spectral function is automatically normalized, allowing for an unambiguous definition of branching ratios via proper integrals over the given line shape. As test cases, we discussed the $\rho (770)$ and $f_0(500)$ resonances, whose pole parameters are known to high precision from dispersive analyses of $\pi \pi$ scattering. In particular, their study allowed us to assess which degrees of freedom are required to capture all relevant features of the respective resonance.

For the $\rho (770)$ we found that a simple Dyson resummation of the self energy, essentially corresponding to a Gounaris–Sakurai parameterization, gives reasonable agreement with phenomenology, but is not sufficient to match the available precision, mainly because the residue is already determined by the pole position. Accordingly, we improved on the construction by including a background term in the two-potential formalism, which provides the required freedom to adjust the residue as well. We observed that the corresponding corrections seem to be concentrated in the energy range in which the excited $\rho '$ and $\rho ''$ resonances appear, and could thus be interpreted as a hint where $4\pi$ effects become relevant [10, 11]. A possible future application concerns the $2\pi$ contribution to hadronic vacuum polarization [8, 12, 13], given that the tensions among different data sets, most prominently BaBar [66], KLOE [67], and CMD-3 [68], indeed appear to point to the study of inelastic effects as an important means to better understand the discrepancies [69].

For the $f_0(500)$, we found that, as expected, it is critical to account for the LHCs, which we implemented including the correct threshold behavior $\propto (-s)^{3/2}$. Moreover, we found that demanding the precise resonance pole position and residue automatically implies a subthreshold zero in the $\pi \pi$ scattering amplitude, which can be naturally identified with the Adler zero. In fact, while it is well known that unitarizing ChPT amplitudes with the Adler zero generates a pole close to the $f_0(500)$ found from Roy equations, our study shows that also the opposite is true. As a by-product, we evaluated the chiral corrections to the position of the Adler zero, finding a $15\%$ reduction compared to its LO value.

Finally, the new way to evaluate spectral functions also allowed us to introduce a new expression to calculate branching fractions via integrals over resonance line shapes that by construction contain information on the correct pole location and residues. We compared our prescription to alternatives proposed in the literature. In some cases significant differences were observed and the origin of those was identified, e.g., related to (lack of) normalization of the spectral function and sensitivity to the line shape far away from the resonance. While the cases we studied are still dominated by the $\pi \pi$ channel, a major advantage of our proposed formalism is that it applies to situations in which different channels can compete, leading to a more complicated analytic structure. This includes the $f_0(980)$, with its strong interplay of $\pi \pi$ and $\bar{K} K$ *S*-waves, as well as the $a_0(980)$, in which case $\pi \eta$ and $\bar{K} K$ have comparable branching fractions. We leave the study of such systems to future work.

## Data Availability

This manuscript has no associated data or the data will not be deposited. [Authors’ comment: The data used in this work can be retrieved from the cited references.]

## Conventions

An integral part of the work presented in this paper concerns the adjustment of parameters to reproduce the correct resonance parameters. Especially the residues are often subject to different conventions and definitions. Here, we collect the various conventions for amplitudes and form factors.

The isoscalar–scalar amplitude on the second sheet close to the pole can be written in the form

A.1 $$\begin{aligned} t^0_0(s)^\text {II} = \frac{g^2 Z \gamma ^\text {II}(s_p)^2}{s_p-s} = \frac{1}{16\pi }\frac{{\tilde{g}_{\sigma \pi \pi }}^2}{s_p-s}\, , \end{aligned}$$

with the renormalization factor *Z*. The form factor can be written in a similar form,

A.2 $$\begin{aligned} F_\pi ^S(s)^\text {II} = \frac{g Z \alpha \gamma ^\text {II}(s_p)}{s_p-s} = \frac{{\tilde{g}_{\sigma \pi \pi }} \sqrt{Z} \alpha }{s_p-s}\, . \end{aligned}$$

We can use this form to write an effective matching condition between the coupling of a scalar source to the $f_0(500)$ and our source coupling $\alpha$. Note that the change from the particle to the isospin basis causes an additional factor in our description, leading to

A.3 $$\begin{aligned} {\langle {0}|}j_S{|{f_{0}(500)}\rangle }&={\langle {0}|}j_S{|{I=0}\rangle }=M_{\pi }^2\tilde{g}_{\sigma S}\nonumber \\&=M_{\pi }^2\sqrt{\frac{3}{2}}\frac{\text {Res}\, F^\text {II}_S}{\tilde{g}_{\sigma \pi \pi }} \nonumber \\&=M_{\pi }^2 \sqrt{\frac{3}{2}}\alpha \sqrt{Z}\, . \end{aligned}$$

The isovector–vector amplitude on the second sheet close to the pole can be written as

A.4 $$\begin{aligned} t^1_1(s)^\text {II}&= \xi ^2(s_p)\frac{g^2 Z \gamma ^\text {II}(s_p)^2}{s_p-s}\nonumber \\&=\frac{(s_p-4M_{\pi }^2)}{3}\bigg [B_{1}\left( \frac{s_p-4M_{\pi }^2}{s_B-4M_{\pi }^2}\right) \bigg ]^2\frac{g^2 Z \gamma ^\text {II}(s_p)^2}{s_p-s}\nonumber \\&= \frac{(s_p-4M_{\pi }^2)}{48\pi }\frac{{\tilde{g}_{\rho \pi \pi }}^2}{s_p-s}\, . \end{aligned}$$

It should be noted that in this case the function *H*(*s*) as defined in Eq. (2.2) absorbs only the squared momentum and the normalization, but not the taming factor. Accordingly, the vector form factor can be written as

A.5 $$\begin{aligned} F_\pi ^V(s)^\text {II}&= B_{1}\left( \frac{s_p-4M_\pi ^2}{s_B-4M_\pi ^2}\right) \frac{g Z \alpha \gamma ^\text {II}(s_p)}{s_p-s}\nonumber \\&= \frac{\alpha \sqrt{Z} {\tilde{g}_{\rho \pi \pi }}}{s_p-s}\, , \end{aligned}$$

leading to

A.6 $$\begin{aligned} \frac{\tilde{g}_{\rho \pi \pi }}{\tilde{g}_{\gamma \rho }}s_p=\alpha \sqrt{Z} {\tilde{g}_{\rho \pi \pi }}\, . \end{aligned}$$

## Electromagnetic channels and couplings

In this work two channels were studied that are different from the massive pseudoscalar ones, $\pi \pi$ and $\bar{K} K$, i.e., the radiative decays into $\gamma \gamma$ and $\pi \gamma$ for the $f_0(500)$ and $\rho (770)$, respectively. The couplings of these channels are suppressed by orders of $\alpha _{\text {em}}$ and the self-energy contributions have a different structure. Here, the definitions of the respective couplings as well as their self-energy contribution are collected.

The width of the decay $f_0\rightarrow \gamma \gamma$ in the narrow-width limit is defined as

B.1 $$\begin{aligned} \varGamma _{f_0\gamma \gamma }=\frac{e^4|\hat{g}_{f_0\gamma \gamma }|^2}{16\pi M_{f_0}} =\frac{|\tilde{g}_{f_0\gamma \gamma }|^2}{M_{f_0}}\text {Im}\,\varPi _{\gamma \gamma }(M_{f_0}^2)\, . \end{aligned}$$

Therefore, the imaginary part on the real axis of the self-energy contribution is given as

B.2 $$\begin{aligned} \text {Im}\,\varPi _{\gamma \gamma }(s)=\frac{1}{16\pi }\, , \end{aligned}$$

which can be used in a once-subtracted dispersion integral to determine explicitly

B.3 $$\begin{aligned} \varPi _{\gamma \gamma }(s)=\frac{1}{16\pi ^2}\log \bigg (\frac{-s_{\gamma \gamma }}{s}\bigg )\, , \end{aligned}$$

where $s_{\gamma \gamma }>0$ is the subtraction point.

The decay $\rho \rightarrow \pi ^0\gamma$ needs to be treated more carefully, since its amplitude reads

B.4 $$\begin{aligned} \mathcal {M}_{\rho \pi \gamma }=e{\hat{g}}_{\rho \pi \gamma }\epsilon _{\mu \nu \alpha \beta }\epsilon _{\rho }^{\mu }\epsilon _{\gamma }^{\nu }p_{\pi }^{\alpha }p_{\gamma }^{\beta }\, . \end{aligned}$$

The width of the decay in the narrow-width limit is defined as

B.5 $$\begin{aligned} \varGamma _{\rho \pi \gamma }&=\frac{e^2|\hat{g}_{\rho \pi \gamma }|^2}{96\pi M_{\rho }^3}(M_{\rho }^2-M_{\pi }^2)^3\nonumber \\&= \frac{|\tilde{g}_{\rho \pi \gamma }|^2}{M_{\rho }}\text {Im}\,\varPi _{\pi \gamma }(M_{\rho }^2)\, , \end{aligned}$$

and therefore

B.6 $$\begin{aligned} \text {Im}\, \varPi _{\pi \gamma }(s)=\frac{1}{16\pi }\frac{s^2}{12}\left( 1-\frac{M_{\pi }^2}{s}\right) ^3 \end{aligned}$$

is used to define the self-energy contribution at the pole by calculating the respective dispersion integral, once-subtracted at $s_0=0$. To tame the energy dependence we use the function $[B_1(x)]^4$, with $B_1(x)$ as defined in Eq. (3.2).

## Two-body left-hand cuts

LHCs in partial-wave amplitudes are due to singularities in crossed (*t*-, *u*-) channels. As they appear as a result of partial-wave projection, the associated integration over the scattering angle in general weakens the singularity: crossed-channel *poles*, e.g., turn into left-hand *cuts*. In the context of crossing-symmetric $\pi \pi$ scattering, the leading LHC is again due to two-pion intermediate states, which lead to a branch point at $s=0$. We discuss the degree of the corresponding singularity with the help of the one-loop function (subtracted at $s=0$)

C.1 $$\begin{aligned} \bar{J}(s)&= 2 - 2\sigma ^2 L_\sigma \,, \quad L_\sigma = \frac{1}{2\sigma }\log \frac{\sigma +1}{\sigma -1} \,, \nonumber \\ \sigma&= \sqrt{1-\frac{4M^2}{s}} = 16\pi \rho (s) \,, \end{aligned}$$

where we have absorbed an overall factor in the definition of $\bar{J}$ for simplicity. Its right-hand cut is of the well-known square-root type,

C.2 $$\begin{aligned} \text {Im}\, \bar{J}(s) = \pi \,\sigma \,\theta \big (s-4M^2\big ) \,. \end{aligned}$$

We now define *s*-channel partial-wave projections of $\bar{J}(t)$ according to

C.3 $$\begin{aligned} \big [P_\ell \bar{J}\big ](s)&\equiv \frac{1}{2}\int _{-1}^1 \text {d}z P_\ell (z) \bar{J}\big (t(s,z)\big ) \,, \nonumber \\ t(s,z)&= \frac{1}{2}\big (4M^2-s\big )(1-z) \,, \end{aligned}$$

which can easily be performed analytically. The imaginary parts of the first few of these are given by

C.4 $$\begin{aligned} \text {Im}\, \big [P_0 \bar{J}\big ](s)&= - \frac{\pi }{\sigma } \big [1-(\sigma ^2-1) L_\sigma \big ]\theta (-s) \,,\\ \text {Im}\, \big [P_1 \bar{J}\big ](s)&= \frac{\pi (1-\sigma ^2)}{2\sigma ^3} \big [1-(\sigma ^2+1) L_\sigma \big ]\theta (-s) \,, \nonumber \\ \text {Im}\, \big [P_2 \bar{J}\big ](s)&= \frac{\pi (1-\sigma ^2)}{4\sigma ^5} \big [\sigma ^2+3-(\sigma ^4+3) L_\sigma \big ]\theta (-s) \,.\nonumber \end{aligned}$$

Near $s=0$, they have the common expansion

C.5 $$\begin{aligned} \text {Im}\, \big [P_\ell \bar{J}\big ](s)&= (-1)^{\ell +1} \frac{2\pi }{3\sigma ^3} + \mathcal {O}\big (\sigma ^{-5}\big ) \\&= (-1)^{\ell +1}\frac{\pi }{12M^3}(-s)^{3/2} + \mathcal {O}\big ((-s)^{5/2}\big ) \,.\nonumber \end{aligned}$$

This can be understood from the fact that the integration for the partial-wave projection hits the LHC first for $z = -1$, where $P_\ell (-1) = (-1)^\ell$. We therefore conclude that left-hand singularities in $\pi \pi$ partial waves are of degree $(-s)^{3/2}$ (only). This is reflected in the form of our conformal parameterization of the background amplitude in Sect. 2.

## Isoscalar *S*-wave Adler zero

In this appendix, we collect the higher-order corrections to the Adler zero in the $\pi \pi$ isospin-0 *S*-wave, $t^0_0(s)$. While the two-loop amplitude has been known since Refs. [70, 71], to the best of our knowledge, even the one-loop corrections to the LO Adler zero

D.1 $$\begin{aligned} s_A^{(2)}=\frac{M_{\pi }^{2}}{2} \end{aligned}$$

have not been spelled out explicitly in the literature despite being used in, e.g., the modified inverse amplitude method [25]. Using $t^0_0(s)$ in the form given in Ref. [72], we find

D.2 $$\begin{aligned} s_A=s_A^{(2)}+s_A^{(4)}+s_A^{(6)}\, , \end{aligned}$$

with

D.3 $$\begin{aligned} s_A^{(4)}&=-\frac{M_{\pi }^{4}}{(48\pi F)^2}\Big [1163+2\big (107 \bar{l}_1+158\bar{l}_2-90 \bar{l}_3\big )\nonumber \\&\quad -908 A-4224 A^2\Big ]\, ,\nonumber \\ s_A^{(6)}&=-\frac{M_{\pi }^{6}}{(12\pi F)^4}\bigg \{\frac{5}{64}\Big (\frac{17948821}{336} + 895 \pi ^2\Big )\nonumber \\&\quad -9A \Big (\frac{349339}{448}+20\pi ^2\Big )\nonumber \\&\quad -\frac{A^2}{7}\Big [\frac{375125}{4}+1008\pi ^2-34776\log \frac{7}{2}\nonumber \\&\quad -6A\big (19073+1296 A\big )\Big ]\nonumber \\&\quad +\frac{4968}{7}\Big [\text {Cl}_3\big (2\sqrt{7} A\big )+2\sqrt{7} A\, \text {Cl}_2\big (2\sqrt{7} A\big ) - \zeta (3)\Big ]\nonumber \\&\quad +\frac{1}{16}\Big [3103\bar{l}_1^2+7364\bar{l}_1\bar{l}_2+4108\bar{l}_2^2\nonumber \\&\quad -2610 \bar{l}_1\bar{l}_3-2340\bar{l}_2\bar{l}_3-1620\bar{l}_3^2\Big ]\nonumber \\&\quad +\frac{1}{896}\Big [ \bar{l}_1\big (1454303-1390328 A-4098432 A^2\big )\nonumber \\&\quad + \bar{l}_2\big (2410997-2315912 A-6820608 A^2\big ) \nonumber \\&\quad -1440\bar{l}_3\big (470-557A-984 A^2\big )\Big ] \nonumber \\&\quad -\frac{L}{128}\Big (21653-12\big (17\bar{l}_1-5257\bar{l}_2+1080\bar{l}_3\big )\Big )\nonumber \\&\quad - 195 L^2+\frac{9}{32}(4\pi )^4 r \bigg \}\, , \end{aligned}$$

where

D.4 $$\begin{aligned} A=\frac{\arctan \sqrt{7}}{\sqrt{7}}\, ,\qquad L=\log \frac{M_{\pi }^{2}}{\mu ^2}\, , \end{aligned}$$

and the Clausen functions are related to polylogarithms via

D.5 $$\begin{aligned} \text {Cl}_3(\theta )=\text {Re}\,\text {Li}_3\big (e^{i\theta }\big )\, , \quad \text {Cl}_2(\theta )=\text {Im}\,\text {Li}_2\big (e^{i\theta }\big )\, . \end{aligned}$$

The $\bar{l}_i$ are given in the conventions of Ref. [73], $M_{\pi }$ is the physical pion mass, and *F* the pion decay constant in the chiral limit. The two-loop low-energy constants are collected in

D.6 $$\begin{aligned} r&=3\big [240 (r_1^r+r_2^r)+428 r_3^r+836 r_4^r+1047 r_5^r+483 r_6^r\big ]\nonumber \\&\simeq -\frac{6F_\pi ^2(3840 f_\chi ^2+896\sqrt{2} f_\chi g_V+147 g_V^2)}{M_V^2}\nonumber \\&\simeq -0.11\, , \end{aligned}$$

where we used the resonance-saturation estimate from Ref. [71] at $\mu =0.77\,\text {GeV}$, with the phenomenological input from Ref. [74]. We neglect uncertainties for the estimate Eq. (D.6), since the impact of *r* on $s_A^{(6)}$ already proves minor, especially in comparison to the dominant uncertainty due to $\bar{l}_1$ in $s_A^{(4)}$. Based on the $\beta$ functions provided in Ref. [75], we also checked that the scale dependence of *r* indeed cancels the one from the *L* and $L^2$ terms in Eq. (D.3). Numerically, we obtain

D.7 $$\begin{aligned} \frac{s_A^{(4)}}{s_A^{(2)}}=-0.12(3)\, ,\qquad \frac{s_A^{(6)}}{s_A^{(2)}}=-0.03(1)\, , \end{aligned}$$

so that in combination $s_A$ reduces by $15(4)\%$ compared to its tree-level value. For the low-energy constants, we used the input $\bar{l}_1=-0.4(6)$, $\bar{l}_2=4.3(1)$ [5], $\bar{l}_3=3.4(3)$, and $F_\pi /F=1.07(1)$ [76–82].

## Footnotes

1 We define the self energy without the coupling, as this allows us to keep track of the parameters appearing in the formalism that are independent of the dynamics.

## Code Availability Statement

This manuscript has no associated code/software. [Authors’ comment: Further details of the numerical analysis can be provided upon reasonable request.]
